# COVID‐19 in Morocco's region: Observational study of prevalence in symptomatic adults using the PANBIOS® rapid antigen test September 2021

**DOI:** 10.1111/irv.13142

**Published:** 2023-05-11

**Authors:** Abbas Ermilo Haroun, Majdouline Obtel, Samia El Hilali, Zhor Zeghari, Najat Oulachguer, Karim Sbai Idrissi, Rachid Razine

**Affiliations:** ^1^ Laboratory of Biostatistic, Clinical and Epidemiological Research, Department of Public Health, Faculty of Medicine and Pharmacy Mohammed V University Rabat Morocco; ^2^ Laboratory of Community Health (Public Health, Preventive Medicine and Hygiene), Department of Public Health, Faculty of Medicine and Pharmacy Mohammed V University Rabat Morocco; ^3^ Municipal Hygiene Office Ministry of Health and Social Protection Rabat Morocco

**Keywords:** community, COVID‐19, rapid antigen PANBIOS®, RT‐PCR, symptomatic

## Abstract

**Background:**

Rapid antigen tests have emerged to deal with the COVID‐19 pandemic. Rapid diagnosis of SARS‐CoV‐2 infection is essential to reduce the spread of the disease. The aim of this study was to estimate the prevalence of COVID‐19 infection and test the sensitivity and specificity in Temara‐Skhirat in symptomatic adults through PANBIOS® test.

**Methods:**

A prospective observational study was conducted in mid‐September 2021. Two investigators conducted data collection from symptomatic adult patients. The diagnostic performance of the PANBIOS®, and the PCR was assessed to calculate sensitivity and the specificity.

**Results:**

Among 206 symptomatic participants, the mean age was 38 ± 12 years, and the majority were women (59%). In our population, 80% had benefited from the anti‐COVID vaccine. The median duration of symptoms was 4 days; the most common symptoms were fatigue (62%), headache (52%), fever (48%), cough (34%), loss of smell (25%), loss of taste (24%), and sore throat (22%). Results revealed 23% of cases tested positive with PANBIOS® test versus 30% with the PCR test. The calculated medical decision between PCR versus PANBIOS® test showed high specificity of 95.7% and a sensitivity of 69.4%. There was concordance between the PANBIOS® test and the PCR.

**Conclusion:**

The prevalence tested remain high, and the sensitivity and specificity of the PANBIOS® versus PCR test are similar to other literatures and close to value described in WHO recommendations. PANBIOS® is a useful test for controlling the spread of COVID‐19 allowing identification of active infection.

## 1 INTRODUCTION

Since first discovered in December 2019, the global pandemic of coronavirus disease 2019 (COVID‐19) caused by the novel coronavirus (SARS‐CoV‐2) poses a serious threat to human life and health.^1^

Morocco, like other countries in the world, has experienced the ups and downs of the coronavirus disease “COVID‐19,” which has caused an unprecedented health crisis. If this pandemic has highlighted points of vulnerability, it has also made it possible to highlight the potentialities that this country holds for addressing the pandemic at different levels. The rapid identification and isolation of symptomatic adult patients within the community has become the cornerstone of controlling the recent outbreak.^2^ Real‐time quantitative polymerase chain reaction (PCR) is commonly used to confirm the diagnosis of COVID‐19 and is considered the gold standard due to its high sensitivity and specificity.^3^ Nevertheless, PCR is labor intensive and time consuming, requires skilled personnel with high cost, and is not available in remote settings.

Antigen tests emerged to address such drawbacks, offering rapid results, an easy‐to‐use procedure, diagnostic focus, and low costs.^4^ In order to prepare against the next season winter, Moroccan's Government decided to introduce rapid antigen test to diagnose COVID‐19 in April 2021. Out of the kit selected the PANBIOS® test manufactured by South Korea. During the second wave of COVID‐19, the Minister of Health in Morocco considers that the rapid antigen tests were not sufficiently tested. So they instructed many national laboratories such as “Pasteur Institut of Casablanca” and National Institute of Hygiene in Rabat to evaluate those rapid antigen tests by showing evidence of their sensitivity and specificity among Morrocans. In mid‐September 2021, viewing the context and the necessity to perform sufficiently the rapid antigen test, we conducted a study according to the World Health Organization. There is an urgent need for rapid and efficient diagnosis of COVID‐19 infection for detection at an early stage, and rapid antigen test provides a direction for referral within a target community, precisely patients with risks factors and symptoms that cannot wait more than 48 h according to the World Health Organization's recommendations.^5^ Early surveillance for viral infections can help control and prevent the spread of infections. In this study, we focus on COVID‐19 infection among symptomatic adult patients, who represent a mobile and dynamic focus of viral transmission. The aim of this study is to estimate the prevalence of COVID‐19 infection and evaluate the sensitivity and specificity in Temara‐Skhirat among symptomatic adults referred by a health center for a rapid PANBIOS® antigen test at a center authorized by the Moroccan Ministry of Health.

## 2 MATERIAL AND METHOD

### 2.1 Study type and population

This is a prospective observational prevalence study conducted at the Temara‐Skhirat center in Morocco during mid‐September 2021.

### 2.2 Inclusion criteria

The patients included in this study were volunteers, having signed an informed consent before inclusion, aged between 18 and 65 years old, symptomatic (general, respiratory, and digestive signs), referred after consultation, and who have performed the rapid antigen test as well as the PCR.

### 2.3 Exclusion criteria

The age groups below 18 and above 65 years of patients were excluded. Asymptomatic patients, contact cases, and seriously ill were also excluded from the study. Inconclusive results and patients who had not benefited from the rapid test as well as the PCR were excluded.

### 2.4 Sampling

The sample was calculated depending on the flow of patients and the national prevalence finds in literature during this period time (about 2 weeks).

The calculation of the sample size was based on the following: Let n be the sample size, p the expected prevalence, e the margin of error at 4% (standard value of 0.04), and t the 95% confidence level (typical value of 1.96). Formula (1) is used to calculate the sample size.^6^

(1) $$n=\left(t^{2}\right)\times p\;\left(1-p\right)/e^{2}.$$

The prevalence of COVID‐19 in adults during this pandemic was made through daily data from the Ministry of Health and with fluctuations due to various factors related to the virus and human behavior. Thus, using this formula, for an expected prevalence of 15%, the number of subjects to be recruited would be approximately 280 subjects. In our study, we performed the rapid test in 206 participants whose plasma was tested with PCR for confirmation.

### 2.5 Data collection

This study was carried out with the authorization of the regional directorate of the Ministry of Health of Rabat‐Salé‐Kenitra, which set up a screening system in a reference center by providing validated PANBIOS® rapid tests and PCR tests in real time.

In partnership with the Department of Public Health of the Faculty of Medicine and Pharmacy of Rabat, two investigators following recommendations of the World Health Organization on the rapid antigenic test participated with the health personnel (doctors and nurses) to support the data collection.

A questionnaire was drawn up with demographic data (age, sex, marital status, profession, educational level, and income), medical history (height, weight, heart disease, high blood pressure, obesity, diabetes, neurological disorder, kidney failure, immunodeficiency, respiratory disease, cancer, anti‐COVID vaccines, and others), and clinical information (COVID‐19, type of contact, symptom duration, fever, cough, fatigue, dyspnea, headache, sore throat, loss of smell, loss of taste, diarrhea, vomiting, nausea, and others) as well as biological tests (antigen test and PCR test).

### 2.6 Management of samples

Using the rapid chromatographic immunoassay technique, the qualitative detection of SARS‐CoV‐2 antigens on nasopharyngeal swabs was performed meticulously by well‐trained residents to avoid any risk to the participant. Interpretation was done within 30 min of the collection. PCR was considered as Gold Standard for participants who benefited from the test.

The transport of the samples from the health center to the National Laboratory (National Institute of Hygiene) was done by health professionals in collaboration with the investigators within 2 h following the sample. The sample was stored at +4°C in a Kombo kits in case of any delay to reach the laboratory; in our knowledge, during the study, all sample reach the National Laboratory in time.

### 2.7 Statistical analysis

A descriptive analysis of the validated data was conducted. Variables were expressed as counts, percentages, means and standard deviations, and medians with interquartile ranges, as appropriate. We performed univariate analysis using parametric and/or non‐parametric statistical tests, as appropriate. We assessed sensitivity and specificity of the PANBIOS**®** and PCR tests. A *p*‐value less than 0.05 was considered statistically significant.

JAMOVI version 1.6 software was used in this statistical analysis.

### 2.8 Ethical considerations

The ethical authorization of the study protocol was obtained from the Ethical Committee (Biomedical Research of the Mohammed V University, Faculty of Medicine and Pharmacy, Faculty of Dental Medicine of Rabat). Its reference number is CERB P‐21.

An information letter and informed consent was also attached to the study protocol.

## 3 RESULTS

**FIGURE 1:**
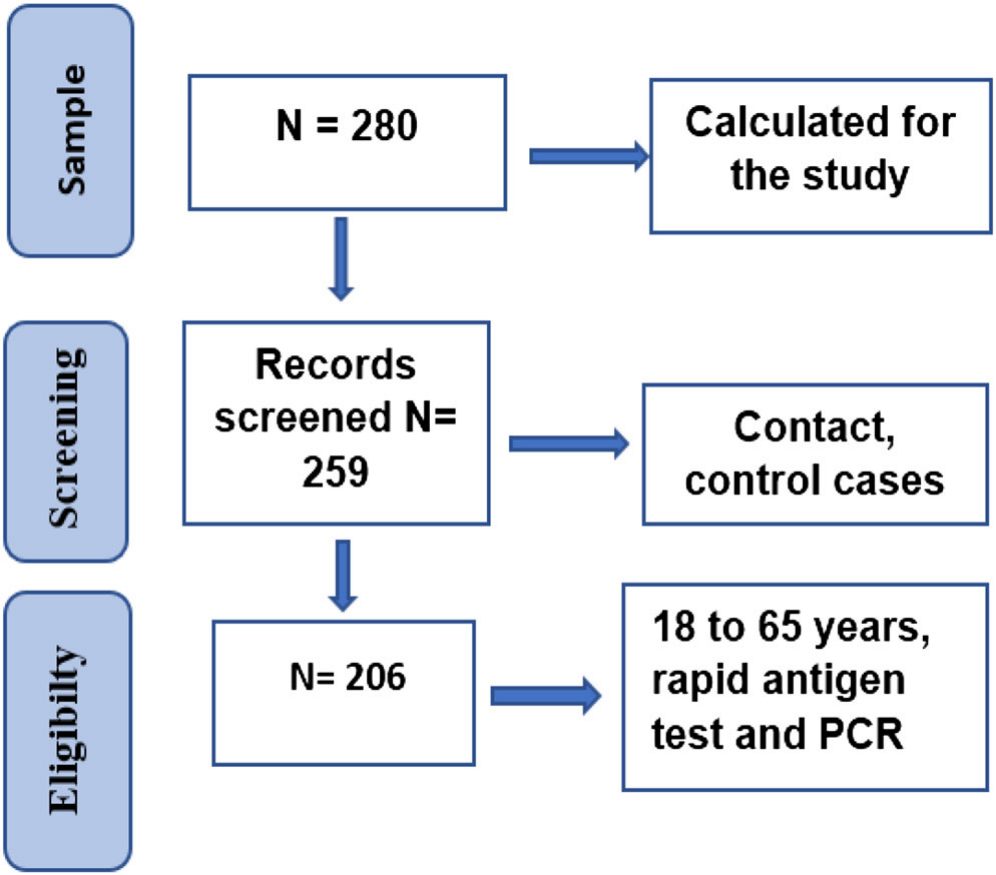
Flow chart.

Of a total of 206 participants (Figure 1), the mean age was 38 years ± 12 standard deviation, and the majority were women (59%). The predominant marital status and occupation were married (60%) and unemployed (44%). Most of the participants had a higher level of education (53%) and an income below 3000 MAD (54%) (Table 1).

**TABLE 1:** Socio‐demographic characteristics of participants in Temara‐Skhirat September 2021.

Characteristics	Values *N* = 206 Counts (percentage %)
Age^a^	38 ± 12
Sex (Female)	123 (59.7)
Marital status	
Single	68 (33.2)
Married	124 (60.5)
Widower	10 (4.9)
Divorce	3 (1.5)
Profession	
Entrepreneur	37 (20.0)
Employee	66 (35.7)
Unemployment	82 (44.3)
Level education	
Illiterate	12 (6)
Primary	18 (9)
Secondary	62 (31.2)
Superior	107 (53.8)
Salary (dirhams)	
Lower 3000	112 (54.6)
Between 3000 and 5000	43 (21.0)
Between 5000 and 10,000	40 (19.5)
More than 10,000	10 (4.9)

a Mean ± deviation.

Around 80% of the participants had benefited from the anti‐COVID vaccine; 77% of the population had been in contact with a family member affected by COVID‐19. The median duration of symptoms was 4 days; the most common symptoms were fatigue (62%), headache (52%), fever (48%), cough (34%), loss of smell (25%), loss of taste (24%), sore throat (22%), and others (diarrhea, nausea, and vomiting) (29%). The results revealed a rate of 23% of positive COVID tests carried out with the rapid PANBIOS® test versus 30% with the PCR test (Table 2).

**TABLE 2:** Clinical and biological characteristics of participants in Temara‐Skhirat September 2021.

Characteristics	Values *N* = 206 Counts (percentage %)
Vaccination against COVID‐19	161 (80)
Past history COVID‐19	24 (12.1)
Type of contact (family)	137 (77)
Heart disease	8 (3.9)
Hypertension	15 (7.3)
Obesity	2 (1)
Diabetes	13 (6.3)
Respiratory disease	3 (1.5)
Others	29 (14.1)
Symptoms duration (day)^a^	4 [3–6]
Fever	97 (48)
Cough	70 (34.5)
Fatigue	127 (62.6)
Dyspnea	29 (14.3)
Headache	106 (52.2)
Sore throat	45 (22.2)
Loss of smell	51 (25.1)
Loss of taste	49 (24.1)
Diarrhea	31 (15.3)
Vomit	8 (4)
Nausea	20 (9.9)
PANBIOS® rapid antigen test (positive)	49 (24.3)
PCR test Gold Standard (positive)	60 (30.8)

^a^ Median [interquartile].

The κ statistic is interpreted as follows: 0 = agreement equivalent to chance; 0.10–0.20 = slight agreement; 0.21–0.40 = fair agreement; 0.41–0.60 = moderate agreement; 0.61–0.80 = substantial agreement; 0.81–0.99 = near‐perfect agreement; and 1.00 = perfect agreement. Negative values indicate that the observed agreement is worse than what would be expected by chance. Also, there was substantial agreement of 0.69 with *p* < 0.001. (Table 3).

**TABLE 3:** Measure of concordance between PANBIOS® and PCR test of participants in Temara‐Skhirat September 2021.

Tests	Kappa (κ)	*p* value	Interpretation
PANBIOS**®** vs. PCR gold standard	0.69	<0.001	Substantial agreement

Table 4 presents the statistical medical decision, a cross table between PCR/PANBIOS**®** rapid antigen test. The results showed a high specificity of 95.7% and a sensitivity of 69.4% with a prevalence of 30.8%.

**TABLE 4:** Sensitivity, specificity, VPP, and VPN of rapid antigen test PANBIOS® and PCR of participants in Temara‐Skhirat September 2021.

Setting	Ratios
	Percentage (%)
Results test antigen PANBIOS**®** vs. PCR gold standard	*N* = 201
Sensitivity	69.4
Specificity	95.7
Predictive positive value	87.8
Predictive negative value	87.5
False negative	30.6
False positive	4.3
Prevalence	30.8

**FIGURE 2:**
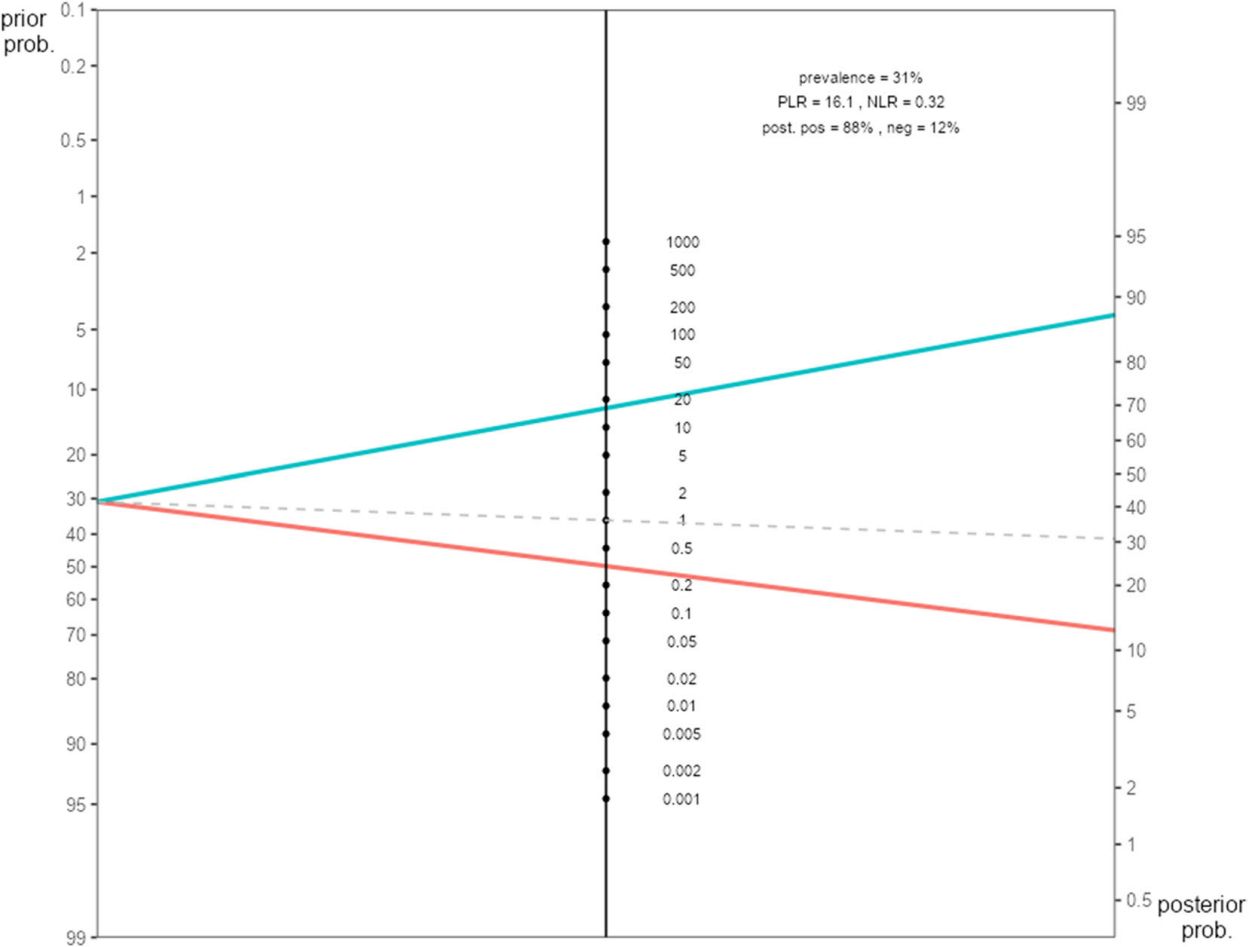
Fagan's nomogram of participants in Temara‐Skhirat September 2021.

The Fagan's nomogram of participants in Temara‐Skhirat September 2021 showed that the probability of a patient having the disease increases from 31% to 88% with a positive test (Figure 2).

## 4 DISCUSSION

In Morocco, rapid antigenic tests have been deployed to deal with the COVID‐19 pandemic. Among these tests, PANBIOS**®** was validated by the Ministry of Health in order to respond quickly to the population's needs and at low cost. It was an urgent request to evaluate and re‐evaluate the diagnostic performance and the agreement of the rapid PANBIOS**®** test given the context of the pandemic. In this study, we estimated the prevalence of COVID‐19 infection in Temara‐Skhirat region among symptomatic young adults referred from a health center for a rapid PANBIOS**®** antigen test. The agreement was substantial with 0.69.^7^

To our knowledge, this is the first study to be carried out at the regional level in Morocco. The results show that the vaccination rate during the study was around 80%.^8^

The results showed that in a symptomatic adult population of 206 patients, there were 49 positive samples or prevalence of 24% using the rapid PANBIOS® test, which is close to the prevalence of the PCR test with 60 positive tests or 30% found in our study, making the PANBIOS**®** rapid test reliable and effective for community‐wide study as a prevalence guidance tool.^9^ Studies have revealed that the rapid PANBIOS**®** test has high sensitivity as recommended by WHO, which estimates a criterion of greater than or equal to 80% for sensitivity and a specificity of 97%.^10^ We found close results, that is, 69% sensitivity and 95% specificity; this difference can be explained by the selection of the population studied or the size of the sample. Indeed, several studies have made comparisons between symptomatic and asymptomatic groups with a larger population.^1^, ^3^ Others studies of symptomatic patients in the Netherlands and Spain found similar test sensitivity (respectively, 72% and 71%) and specificity (respectively, 100% and 99%).^11^, ^12^ In contrast, a study made in Ethiopia shows better sensitivity 99.6% and a specificity of 99.1% using Bayesian latent‐class models.^13^ This difference could be explained by the size of our sample and the fact that we only specify symptomatic patients. Another hypothesis could be the methods used, we used different tools to evaluate the performance of the rapid PANBIOS®, and a medical decision using JAMOVI was made.^14^

The rapid PANBIOS**®** and PCR test is associated (*p* < 0.001), and similar results on sensitivity and specificity have been described in an article.^15^

Our study showed limits, and we found 4% false negative and 30% false positive in symptomatic patients; this difference may be due to several factors, the early or late onset of COVID‐19 disease; indeed, studies have shown that the rapid PANBIOS® test was more effective during the first 5 days after disease onset and the number of false negative tests in RDT increases with time after the onset of clinical symptoms and low Ct value, especially after more than 1–2 weeks.^13^, ^16^, ^17^, ^18^, ^19^

In some cases, however, antigen tests can give false‐positive results: The result is positive when there is no viral infection. According to Jac Dinnes, “on average we see false positive rates of only 2–3%.”^20^ Among the scenarios causing a false positive, we note the mishandling of the tests, the defectiveness of certain batches, or even the reading of the test before or after the time recommended by the manufacturer. The PCR test was conducted in the National Institut Laboratory, and results were communicated to the department. During these scenarios, many could have cause this high false‐positive founds in our results. One of the scenario could be a mismanipulation of the PCR test by the technician or a basis of confusion during communication of the results as indetermined cases were not counted as positive because of their clinical symptoms.

Sensitivity and specificity increased in symptomatic patients. For a prevalence of 30% using the medical decision test, we have 88% positive test and 12% negative test.

## 5 CONCLUSION

The prevalence of COVID‐19 remains high in Temara‐Skhirat region, Morocco. The PANBIOS**®** rapid test is a quick diagnostic orientation tool for COVID‐19 for a community screening campaign. PCR should not be dismissed as a Gold Standard in case of doubt or suspicion of false negatives. The COVID‐19 pandemic has shown the weaknesses of Morocco's health system and the need of a new diagnostic strategy. Our research brings attention to the improvement of diagnostic tools and access of the latter to all in need. PANBIOS**®** is a useful test for controlling the spread of COVID‐19 allowing identification of active infection and isolation of positive patients.

### 5.1 Benefits of the study

This study estimated the seroprevalence of COVID‐19 and identified certain risk factors among the adult population of Temara‐Skhirat during the current pandemic. The study also highlighted a possible comparator threshold between the rapid and PCR tests in early COVID‐19 detection. Finally, the study informed decision makers regarding the applicability of rapid detection tools to better control this epidemic for mass gathering.

## AUTHOR CONTRIBUTIONS

*Data collection*: Abbas Ermilo Haroun and Samia El Hilali. *Formal analysis*: Abbas Ermilo Haroun. *Investigation*: Abbas Ermilo Haroun and Samia El Hilali. *Methodology*: Abbas Ermilo Haroun, Samia El Hilali, Rachid Razine, and Majdouline Obtel. *Supervision*: Majdouline Obtel, Najat Oulachguer, Karim Sbai Idrissi, and Rachid Razine. *Writing—original draft*: Abbas Ermilo Haroun and Samia El Hilali. *Writing—review and editing*: Abbas Ermilo Haroun, Samia El Hilali, Zhor Zeghari, Najat Oulachguer, Karim Sbai Idrissi, Majdouline Obtel, and Rachid Razine. All authors have read and agreed to the published version of the manuscript.

## CONFLICT OF INTEREST STATEMENT

The authors declare that they have no conflict of interest.

### PEER REVIEW

The peer review history for this article is available at https://www.webofscience.com/api/gateway/wos/peer-review/10.1111/irv.13142.

## Data Availability

The data are available for the manuscript.
